# Myocardial iron overload by cardiovascular magnetic resonance native segmental T1 mapping: a sensitive approach that correlates with cardiac complications

**DOI:** 10.1186/s12968-021-00765-w

**Published:** 2021-06-14

**Authors:** Antonella Meloni, Nicola Martini, Vincenzo Positano, Antonio De Luca, Laura Pistoia, Sara Sbragi, Anna Spasiano, Tommaso Casini, Pier Paolo Bitti, Massimo Allò, Paola Maria Grazia Sanna, Raffaele De Caterina, Gianfranco Sinagra, Alessia Pepe

**Affiliations:** 1 Magnetic Resonance Imaging Unit, Fondazione G. Monasterio CNR-Regione Toscana, Pisa, Italy; 2 Cardiovascular Department, University of Trieste, Trieste, Italy; 3 Cardiovascular Division, University of Pisa, Pisa, Italy; 4 Unità Operativa Semplice Dipartimentale Malattie Rare del Globulo Rosso, Azienda Ospedaliera di Rilievo Nazionale “A. Cardarelli”, Napoli, Italy; 5 Centro Talassemie ed Emoglobinopatie, Ospedale “Meyer”, Firenze, Italy; 6 Servizio Immunoematologia e Medicina Trasfusionale, Dipartimento dei Servizi, Presidio Ospedaliero “San Francesco” ASL Nuoro, Nuoro, Italy; 7 Ematologia Microcitemia, Ospedale San Giovanni di Dio, ASP Crotone, Crotone, Italy; 8 Servizio Trasfusionale Aziendale, Azienda Ospedaliero-Universitaria di Sassari, Sassari, Italy

**Keywords:** Iron overload, Thalassaemia major, Magnetic resonance imaging, Cardiovascular diseases

## Abstract

**Background:**

We compared cardiovascular magnetic resonance segmental native T1 against T2* values for the detection of myocardial iron overload (MIO) in thalassaemia major and we evaluated the clinical correlates of native T1 measurements.

**Methods:**

We considered 146 patients (87 females, 38.7 ± 11.1 years) consecutively enrolled in the Extension-Myocardial Iron Overload in Thalassaemia Network.

T1 and T2* values were obtained in the 16 left ventricular (LV) segments. LV function parameters were quantified by cine images. Post-contrast late gadolinium enhancement (LGE) and T1 images were acquired.

**Results:**

64.1% of segments had normal T2* and T1 values while 10.1% had pathologic T2* and T1 values. In 526 (23.0%) segments, there was a pathologic T1 and a normal T2* value while 65 (2.8%) segments had a pathologic T2* value but a normal T1 and an extracellular volume (ECV) ≥ 25% was detected in 16 of 19 segments where ECV was quantified.

Global native T1 was independent from gender or LV function but decreased with increasing age. Patients with replacement myocardial fibrosis had significantly lower native global T1. Patients with cardiac complications had significantly lower native global T1.

**Conclusions:**

The combined use of both segmental native T1 and T2* values could improve the sensitivity for detecting MIO. Native T1 is associated with cardiac complications in thalassaemia major.

## Background

Iron-induced cardiomyopathy is the leading cause of death in β-thalassaemia major (β-TM) patients [1]. Myocardial iron overload (MIO) can be removed or prevented by chelation therapy [2], which should be tailored to the individual patient needs. Thus, techniques for assessing MIO and its response to chelation therapy are essential for the best management of β-TM patients.

To date, cardiovascular magnetic resonance (CMR) is the only non-invasive technique for quantification of MIO. The presence of myocardial iron deposits causes microscopic magnetic field inhomogeneities and results in a reduction in T1, T2 and T2* relaxation times [3]. The T2* technique, introduced in 1999 [4], is currently the method of choice for cardiac iron quantification and it represents the first parametric mapping technique to become a clinical tool and a reference method according to clinical guidelines [5]. The T2* technique is fast, robust, reproducible, transferable among different CMR scanners [6–8], and validated against tissue in human models [9, 10]. The deployment of T2* CMR has been recognized as one of the main drivers for the improvement in survival of TM patients, leading to the reduction in deaths due to MIO [1]. In fact, cardiac T2* values have a prognostic role, allowing the early identification of patients at risk for heart failure and needing an intensification of chelation therapy [11, 12]. However, the T2* technique is sensitive to susceptibility artifacts and has reduced sensitivity for detection of changes associated with mild or early MIO [13].

In vivo mapping of the myocardial T1 relaxation time has been proposed as a possible approach to overcome this limitation. A significant correlation between mid-septum native T1 and T2* values has been detected in patients with β-TM and other haemoglobinopathies [14–18].

No literature data are available about the association between native T1 and T2* using a segmental approach including the entire left ventricle (LV). As previously demonstrated for the T2* technique, the segmental approach is more sensitive, allowing to detect MIO in the various cardiac segments before it occurs at the septal level [19]. This approach is particularly valuable in young patients [20] and allows the detection of preferential patterns of iron distribution correlated with clinical endpoints [12, 21].

To date, no study has evaluated whether T1 measurements quantifying MIO are influenced by age, gender, or myocardial fibrosis. Of note, replacement myocardial fibrosis detected by late gadolinium enhancement (LGE) CMR is common in β-TM patients [22] and it is associated with an higher risk of cardiac complications [12]. By preliminary experience extracellular volume (ECV) appears to be increased in TM and associated with MIO [23]. Finally, the clinical impact of T1 for detecting cardiac complications in β-TM has never been explored.

We sought to assess the relationship between native T1 and T2* values using a segmental approach, as well as the association between global T1 measurements, demographics, and cardiac involvement.

## Methods

### Study population

We considered 146 β-TM patients (87 females, 38.7 ± 11.1 years), consecutively enrolled in the Extension-Myocardial Iron Overload in Thalassaemia (E-MIOT) project, an Italian network constituted by 66 thalassaemia centres and 11 validated CMR sites [8, 24, 25] sharing clinical data.

The study complied with the Declaration of Helsinki and was approved by the local ethical committee. All patients gave written informed consent.

### CMR protocol

CMR exams were performed in the reference CMR center of the E-MIOT Network (Pisa) using a 1.5 T CMR scanner (Signa Artist; General Electric Healthcare, Waukesha, Wisconsin, USA). A 30-element cardiac phased-array receiver surface coil with breath-holding and electrocardiographic (ECG)-gating was used.

For T1 mapping, three parallel short-axis slices (basal, medium and apical) of the LV were acquired in end-diastole using a modified Look-Locker inversion recovery (MOLLI) sequence [26] with a 3 (3 s) 3 (3 s) 5 scheme. Other sequence parameters were: slice thickness = 8 mm, flip angle = 35°, matrix = 172 × 172 pixels, partial Fourier = 0.75. Starting from the 11 motion-corrected images with different inversion times, pixel-wise T1 maps were generated on the scanner using a three-parameter fitting model [26]. T1 maps were transferred to a dedicated workstation for offline post-processing, that involved manual tracing of endocardial and epicardial borders, with care taken to avoid blood pool and epicardial fat. Basal and medium slices were divided into 6 equiangular segments and the apical slice in 4 segments, according to the American Heart Association/American College of Cardiology model [27]. The T1 in each segment was obtained by averaging the T1 value for all the pixels within the segment. For blood analysis, a region was drawn in the LV blood pool of each short axis slice avoiding papillary muscles.

T2* multiecho images were acquired in the same 3 slice locations used for T1 mapping by a multi-echo gradient-echo T2* sequence (10 echo times-TEs). Sequence parameters and T2* images analysis, performed using a custom-written and validated software (HIPPOMIOT^®^), have been already described [19]. After the calculation of T2* values in all myocardial segments, an appropriate correction map compensated for cardiac/visceral geometrical and susceptibility artifacts [28].

Global T1 and T2* values were obtained by averaging all segmental T1 and T2* values, respectively. Mid-septum T1 and T2* values were obtained by averaging the values in segments 8 and 9.

Balanced steady-state free precession cine images were acquired in sequential 8-mm short-axis slices from the atrio-ventricular ring to the apex to quantify LV function parameters in a standard way using MASS^®^ software (Medis, Leiden, The Netherlands) [29]. LV volumes and mass were normalized for the body surface area. Segmental wall motion was visually assessed in cine images.

To detect the presence of replacement myocardial fibrosis, LGE short-axis, vertical, horizontal, and oblique long-axis images were acquired 10–18 min after gadobutrol (Gadovist^®^; Bayer Healthcare; Berlin, Germany) intravenous administration (0.2 mmoL/kg). LGE images were not acquired in patients with an estimated glomerular filtration rate < 30 mL/min/1.73 m^2^ and in patients who declined contrast. LGE was considered present when visualized in two different views [22, 30].

Post-contrast T1 images were acquired 10 min after contrast medium administration and image analysis was performed by using the same approach employed for native T1 images. Segmental ECV values were calculated with input of native and post-contrast myocardial segmental and blood pool T1 values and same-day hematocrit [31].

### Diagnostic criteria

The lower limit of normal T1 and T2* for each segment was established as mean-2 standard deviations (SD) on data acquired on 80 healthy subjects. Since in healthy subjects T1 values are influenced by gender, gender-specific segmental thresholds were derived.

Heart failure (HF) was diagnosed by clinicians based on symptoms, signs and instrumental findings [32]. All arrhythmias were ECG-documented and required specific medication [33]. The term “cardiac complications” included HF and arrhythmias.

### Reproducibility analysis for T1 measurements

To evaluate the intra-observer reproducibility, native T1 images from 20 patients were re-analyzed by the same operator after 4 weeks. To evaluate the inter-observer reproducibility, the same images were blindly analyzed by a second operator. The coefficient of variation (CoV) was calculated as the ratio of the SD to the mean, multiplied by 100. The intraclass correlation coefficient (ICC) was obtained from a two-way random effects model with measures of absolute agreement. The Bland–Altman technique was used to plot the absolute difference versus the average values between the two datasets. Bias was the mean of the difference between the two sets of values and agreement was the mean ± 1.96 SDs.

### Statistical analysis

All data were analyzed using SPSS (version 18.0, Statistical Package for the Social Sciences, International Business Machines, Inc., Armonk, New York, USA).

Continuous variables were described as mean ± SD and categorical variables were expressed as frequencies and percentages.

The Kolomogorov-Smirnov test showed a non-normal distribution for T1 values.

The comparison of T1 values between two groups was made by the Wilcoxon rank sum test. χ^2^ testing was performed for non-continuous variables.

Correlation analysis was performed using the Spearman’s test.

Logistic regression was used to evaluate the odds ratio (OR) with 95% confidence intervals (CI). The OR was used to compare the odds for two groups.

To determine the best cut-off of T1 or T2* values for discriminating the presence of a specific condition, the maximum sum of sensitivity and specificity was calculated from receiver-operating characteristic (ROC) curve analysis.

A 2-tailed P < 0.05 was considered statistically significant.

## Results

### Patients’ characteristics

Patients’ demographic and clinical characteristics are summarized in Table 1.

**Table 1:** Demographic, clinical and cardiovascular magnetic resonance (CMR) data of the patients

Variable	Value
Sex (males/females)	59/87
Age (years)	38.7 ± 11.1
Transfusion starting age (months)	16.5 ± 14.9
Chelation starting age (years)	4.5 ± 5.6
Splenectomy, %	40.6
Past or active hepatitis C virus infection, (%)	67.2
Pre-transfusion hemoglobin (g/dl)	9.7 ± 0.4
Serum ferritin (ng/l)	1205 ± 1147
Global myocardial T1 (ms)	952 ± 99
Global myocardialT2*(ms)	37.4 ± 9.3
LV end-diastolic volume index (ml/m^2^)	83.8 ± 16.4
LV end-systolic volume index (ml/m^2^)	31.7 ± 10.3
LV stroke volume index (ml/m^2^)	52.0 ± 9.9
LV mass index	59.6 ± 12.8
LV ejection fraction (%)	62.5 ± 7.4
Abnormal LV motion, N (%)	14 (9.6)
Replacement myocardial fibrosis, N (%)	36/88 (40.9%)

*LV* left ventricular, *N* number

Mean global heart T1 value was 952 ± 99 ms (range 494–1170 ms) while mean global heart T2* value was 37.4 ± 9.3 ms (range 5.2–37.4 ms).

**Fig. 1:**
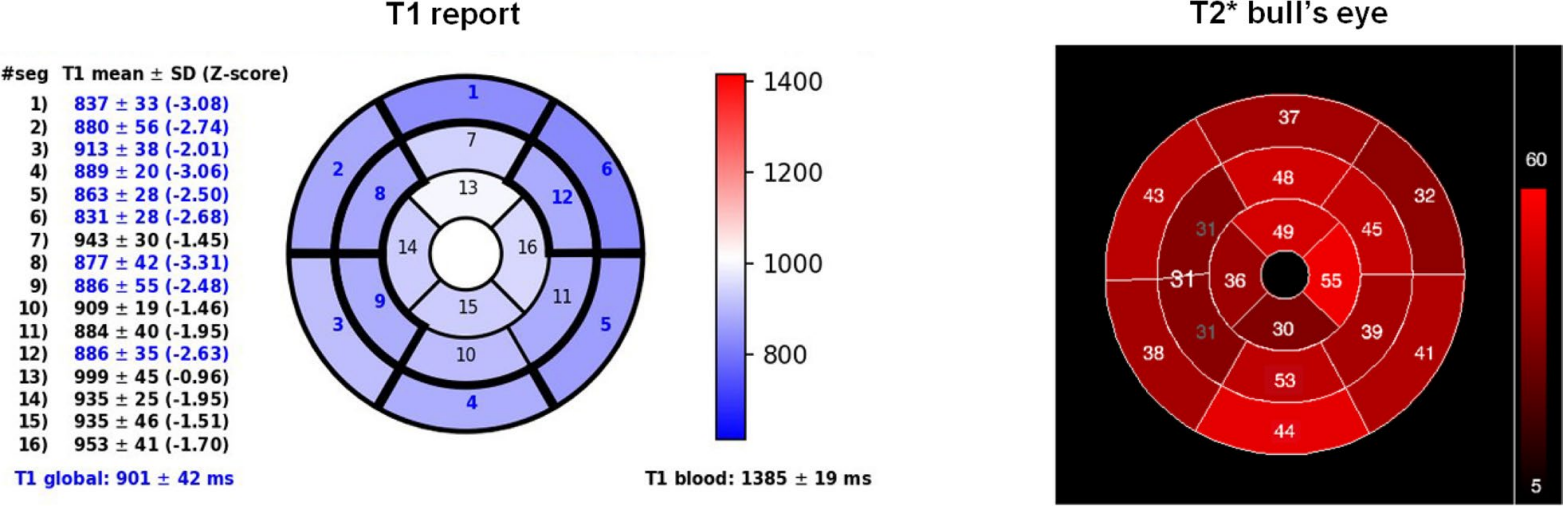
Representative myocardial native T1 and T2* reports for a patient with pathologic segmental T1 but normal T2*

Figure 1 shows T1 and T2* reports for one patient.

### Corrrelation between global heart and mid-septum T1 and T2* values

A significant correlation was detected between global heart and mid-septum T1 values (R = 0.914; P < 0.001). Out of the 70 patients with a reduced global heart T1 value (< 928 ms in males and < 989 ms in females), 58 (82.0%) had also a reduced mid-septum T1 value (< 919 ms in males and < 966 ms in females) while 12 (17.1%) had a normal mid-septum T1 value. Furthermore, out of the 85 patients with a normal mid-septum T1 value, 44 (51.8%) showed a pathological T1 value in at least one of the remaining 14 segments. For three patients a reduced mid-septum T1 value was detected in presence of a normal global heart T1 value, but all of them had also other two segments with reduced T1 value.

For T2* a significant correlation was detected between global heart and mid-septum values (R = 0.733; P < 0.001). Four patients had a normal mid-septum T2* value in presence of a reduced global heart T2* value and out of the 127 patients with a normal mid-septum T2* value, 35 (27.6%) showed a pathological T1 value in at least one of the remaining 14 segments.

### Intra- and inter-observer variability

The results of the intra- and inter-observer variability analysis for segmental and global T1 values are indicated in Table 2. The CoV was below 5% for all segments and the ICC was always excellent (> 0.90). The Bland–Altman analysis demonstrated no significant bias.

**Table 2:** Intra- and inter-observer reproducibility data for T1 measurements

	Intra-observer				Inter-observer			
	CoV (%)	ICC (95% CI)	Bland–Altman analysis		CoV (%)	ICC (95% CI)	Bland–Altman analysis	
			Bias (ms)	Limits (ms)			Bias (ms)	Limits (ms)
Segment 1	1.45	0.997 (0.993–0.999)	− 2.7	− 35.6–39.2	4.83	0.996 (0.991–0.999)	4.6	− 33.1–42.2
Segment 2	2.24	0.992 (0.979–0.997)	1.7	− 51.5–54.8	2.09	0.992 (0.980–0.997)	9.9	− 36.1–55.8
Segment 3	1.17	0.998 (0.994–0.999)	3.3	− 23.7–30.4	2.42	0.991 (0.977–0.996)	5.6	− 50.9–62.2
Segment 4	1.62	0.996 (0.989–0.998)	0.1	− 39.1–39.3	1.82	0.994 (0.984–0.998)	9.8	− 29.7–49.3
Segment 5	1.45	0.995 (0.987–0.998)	− 4.1	− 37.9–29.7	3.10	0.977 (0.942–0.991)	8.4	− 64.3–81.1
Segment 6	1.81	0.994 (0.985–0.998)	0.6	− 41.3–42.5	2.79	0.986 (0.961–0.995)	13.6	− 45.5–72.6
Segment 7	2.42	0.992 (0.981–0.997)	7.6	− 49.3–64.5	3.83	0.979 (0.948–0.992)	12.7	− 77.8–103.1
Segment 8	1.38	0.997 (0.992–0.999)	− 6.9	− 36.8–23.0	1.79	0.995 (0.975–0.999)	13.8	− 19.2–46.9
Segment 9	1.32	0.998 (0.994–0.999)	− 6.3	− 34.5–21.9	3.29	0.986 (0.965–0.995)	14.5	− 57.4–86.4
Segment 10	1.59	0.997 (0.992–0.999)	− 5.0	− 41.4–31.4	2.49	0.992 (0.981–0.997)	5.9	− 52.2–63.9
Segment 11	1.77	0.9954 (0.986–0.998)	5.3	− 36.7–47.3	1.79	0.994 (0.985–0.998)	− 4.7	− 47.8–38.4
Segment 12	1.74	0.994 (0.986–0.998)	3.7	− 38.9–46.3	2.93	0.984 (0.960–0.994)	10.2	− 60.4–80.8
Segment 13	2.50	0.985 (0.962–0.994)	-8.2	− 75.2–58.9	2.13	0.989 (0.970–0.996)	11.7	− 42.2–65.9
Segment 14	1.52	0.996 (0.989–0.998)	2.8	− 36.3–41.9	2.55	0.987 (0.964–0.995)	14.9	− 44.7–74.5
Segment 15	1.90	0.994 (0.985–0.998)	3.0	− 45.5–51.4	3.19	0.983 (0.957–0.993)	− 1.5	− 83.4–80.4
Segment 16	2.61	0.985 (0.963–0.994)	− 0.1	− 74.0–73.9	2.22	0.988 (0.971–0.995)	− 5.7	− 67.3–56.0
Global	0.70	0.999 (0.998–1.000)	− 0.3	− 17.7–17.0	1.35	0.997 (0.989–0.999)	7.7	− 21.7–37.2

*CoV* coefficient of variation, *ICC* intraclass correlation coefficient

### Correlation between T1 and T2* values by a segmental approach

T1 images showed more pronounced motion artifacts and lower contrast-to-noise-ratio, determining the exclusion of 49/2336 (2.1%) segments. No segments were excluded in T2* images. Globally 2287 segmental T1 and T2* values were considered.

**Fig. 2:**
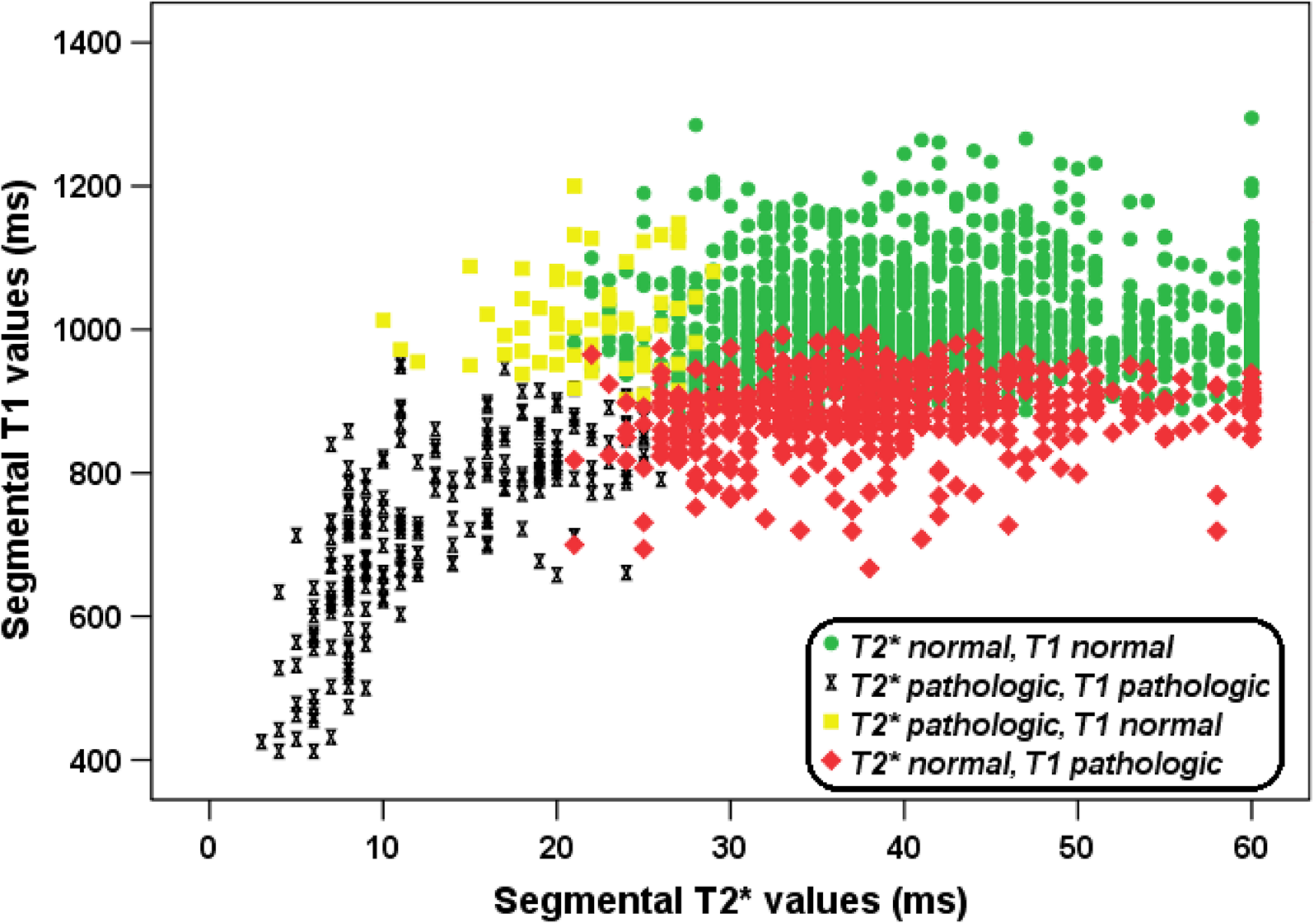
Scatter plot showing the relationship between segmental T1 and T2*

Figure 2 shows the relationship between segmental T1 and T2* values. For patients with pathologic segmental T2* values there was a linear relationship between segmental T1 and T2* values (R = 0.775, P < 0.001). Normal T2* and T1 values were found in 465 (64.1%) segments while 231(10.1%) segments had pathologic T2* and T1 values, giving a concordance between the two measurements of 74.2%. For 526 (23.0%) segments (95 patients) a pathologic T1 value was detected in the presence of a normal T2* value. For 65 (2.8%) segments (33 patients) a pathologic T2* value was detected in the presence of a normal T1 value. ECV was measured in 19 of 65 segments with pathologic T2* but normal T1 value (12 patients) and an ECV ≥ 25% was detected in 16 (84.2%) segments. LGE images were available for 30/65 segments (18 patients) and only one segment showed LGE-positivity. This single segment had also increased ECV.

A significant correlation was detected between global heart T1 and T2* values (R = 0.449; P < 0.001). Out of the 22 (15.1%) patients with a pathologic global heart T2* value (< 32 ms), two had a normal T1. Fifty (34.2%) patients had a pathologic global T1 in the presence of a normal global T2*.

### Global T1 and demographics

Myocardial global T1 was comparable between males and females (944 ± 90 vs. 957 ± 104 ms; P = 0.073) while they showed a weak inverse correlation with age (R = − 0.220; P = 0.007).

A significant inverse correlation was detected between global T1 and serum ferritin levels (R = − 0.297; P = 0.009).

### Global heart T1 values and CMR findings

No correlation was detected between global T1 and LV volume indexes, mass index, or ejection fraction.

Abnormal LV motion  was found in 14 (9.6%) patients: 13 hypokinetic and one dyskinetic. Patients with abnormal LV motion   had significantly lower global T1 than patients without LV motion abnormalities  (884 ± 140 vs. 959 ± 91 ms; P = 0.049). At ROC curve analysis, a global T1 ≤ 865 ms predicted the presence of LV motion abnormalities with a sensitivity of 42.9% and a specificity of 90.2% (P = 0.049). The area under the curve (AUC) was 0.66 (95% Confidence interval: 0.58–0.74).

**Fig. 3:**
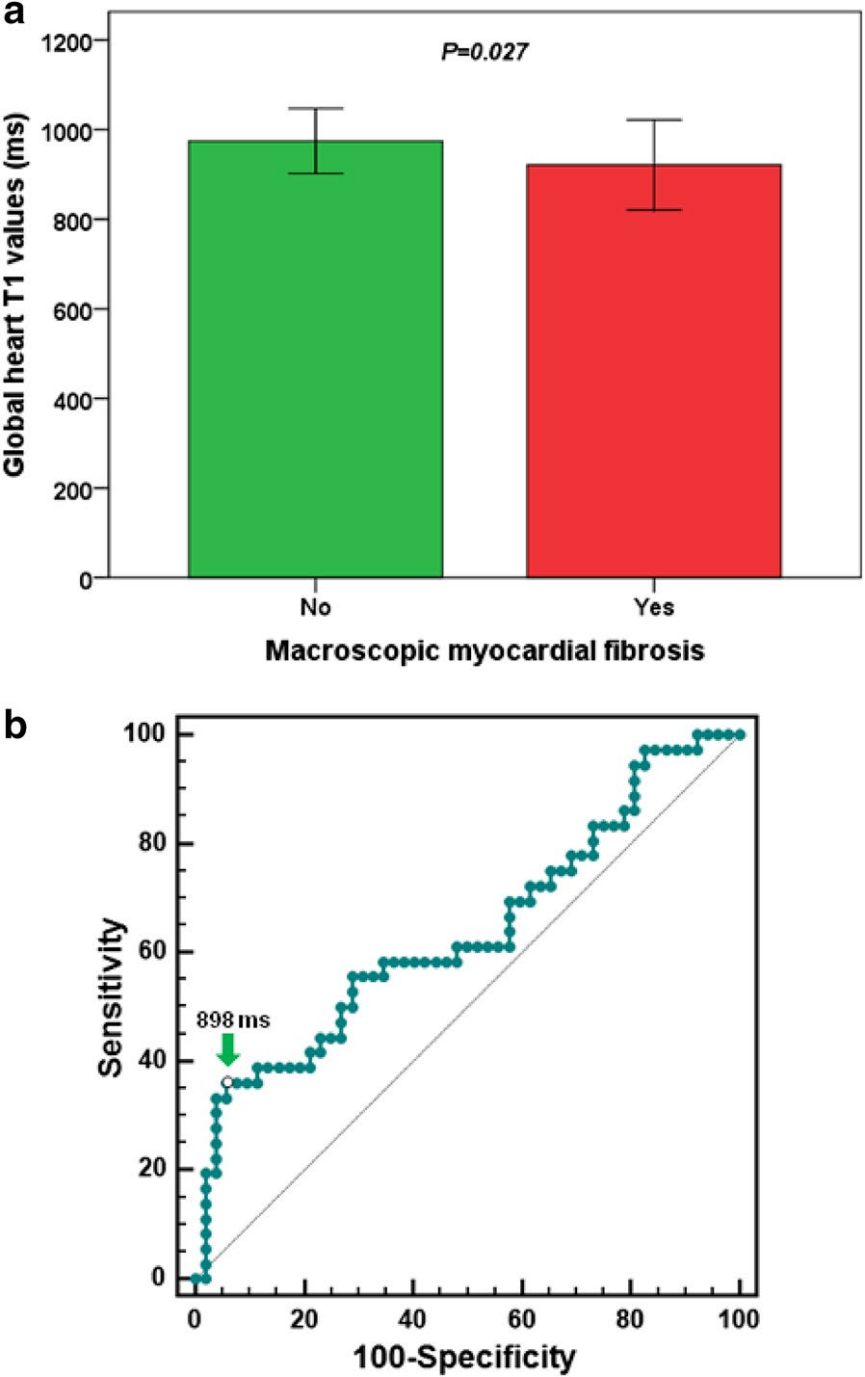
**a** Global T1 values in patients without and with replacement myocardial fibrosis detected by late gadolinium enhancement (LGE). **b** ROC curve analysis of global heart T1 values to predict LGE positivity

LGE images were acquired in 88 (60.3%) patients; replacement myocardial fibrosis was detected in 36 (40.9%) patients. Two patients showed an ischemic pattern. Among the patients with LGE, 72.2% had two or more foci of fibrosis. Patients with replacement myocardial fibrosis had significantly lower global T1 (921 ± 100 vs. 975 ± 73 ms; P = 0.027) (Fig. 3a) and T2* values (34.2 ± 11.5 ms vs. 39.2 ± 6.9 ms; P = 0.036). At ROC curve analysis, a global heart T1 value ≤ 898 ms predicted the presence of positive LGE with a sensitivity of 36.1% and a specificity of 94.2% (P = 0.026). The AUC was 0.64 (95% Confidence interval: 0.53–0.74) (Fig. 3b).

LGE was present for 1408 segments (88 patients x16 segments) and 105 (7.5%) were positive. Segments with LGE had significantly lower T1 than segments LGE-negative (906 ± 111 ms vs. 957 ± 104 ms; P < 0.001).

### T1 values and cardiac complications

**Fig. 4:**
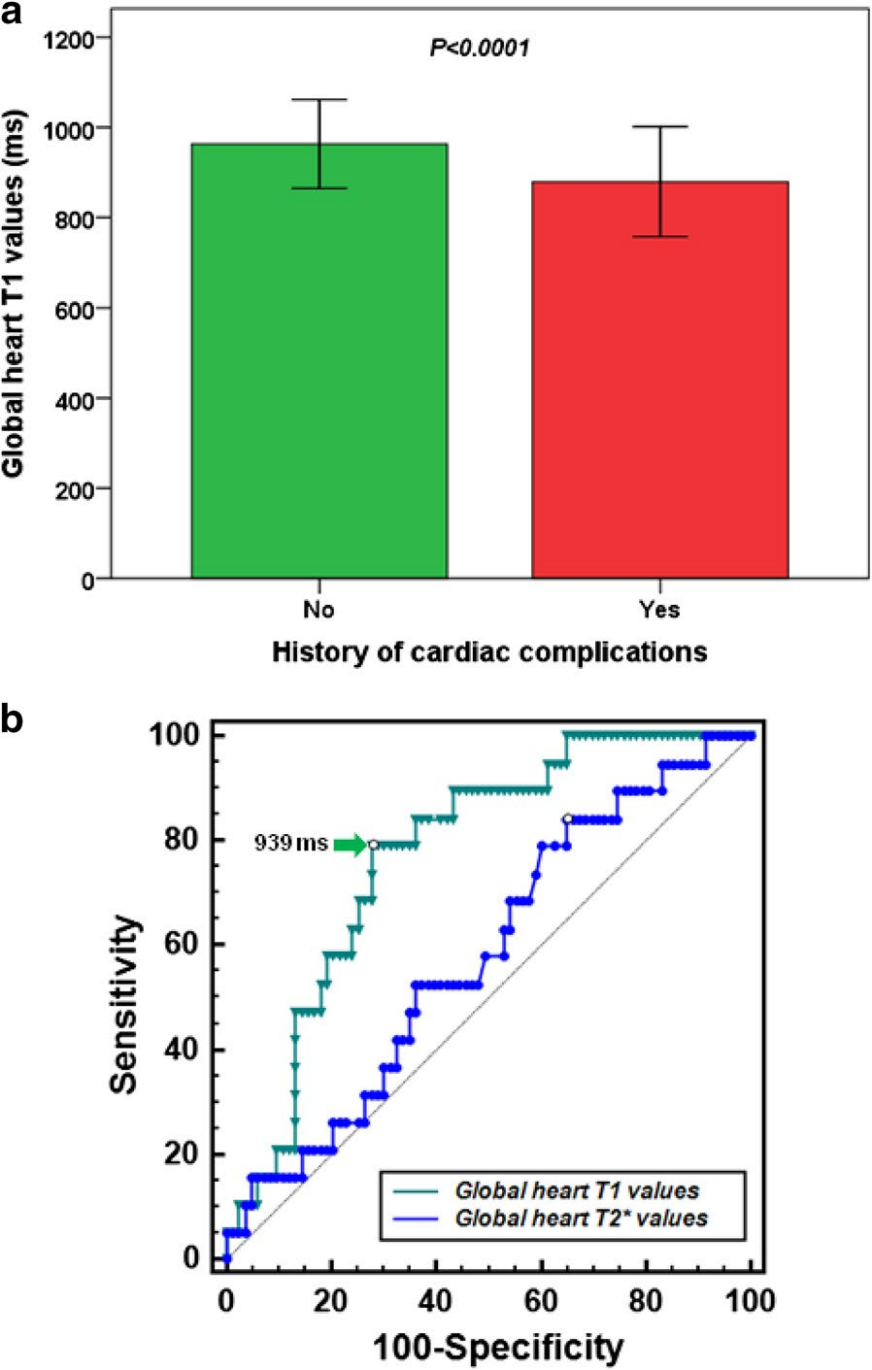
**a** Global myocardial T1 in patients without and with history of cardiac complications. **b** ROC curve analysis of global T1 (green) and T2* (blue) to identify a positive history of cardiac complications

Nineteen patients had an history of cardiac complications: 11 HF and 8 arrhythmias (7 supraventricular and 1 ventricular). Patients with cardiac complications had significantly lower global T1 (874 ± 122 vs. 962 ± 99 ms; P < 0.001) (Fig. 4a) but similar T2* (33.9 ± 11.7 vs. 36.9 ± 9.3 ms; P = 0.260). Cardiac complications were more frequent in the group of patients with reduced versus normal global T1 (29.8% vs. 9.1%; P = 0.010). OR for cardiac complications was 4.2 (95%CI = 1.4–12.9) for patients with reduced global T1 versus patients with normal global T1.

At ROC curve analysis, a global T1 ≤ 939 ms predicted a positive history of cardiac complications with a sensitivity of 78.9% and a specificity of 72.3% (P < 0.001). The AUC was 0.77 (95% confidence interval: 0.68–0.85). The ROC curve did not reveal a global heart T2* threshold below which the probability of detecting a positive history of cardiac complications increases significantly with satisfying sensitivity and specificity (AUC = 0.58; P = 0.238). The Delong’s test showed a significant difference among the AUCs (P = 0.005) (Fig. 4b).

The optimal cut-off for the mid-septum T1 value was 957 ms (P < 0.001) and the AUC was 0.70 (95% Confidence interval: 0.66–0.83). No significant difference (P = 0.449) was obtained between the AUCs obtained for global and mid-septum T1.

For eight patients, the cardiac complication was still active at the time of the CMR (6 supraventricualr arrhythmias and 2 HF). These patients had a lower T1 than the group of patients without history of cardiac complications or with resolved cardiac complications at the time of CMR (855 ± 153 vs. 954 ± 101 ms; P = 0.012) but comparable global heart T2* values (34.2 ± 12.3 vs. 36.6 ± 9.6 ms; P = 0.422).

## Discussion

To our knowledge, this is the first report relating native T1 to T2* values by a segmental approach including the entire LV wall in haemochromatotic patients. The segmental approach allows to identify a heterogeneous and early iron distribution, that would remain otherwise unexplored with a single measurement in the mid-ventricular septum [10, 19, 34]. For both T1 and T2*, although we found a significant correlation between global and mid-septum values, the segmental approach was demonstrated to be more sensitive. For T2* mapping the clinical advantages of the more sensitive segmental technique have been demonstrated in order to have an iron-free patient [12, 19–21, 34, 38, 43]. For T1 mapping larger and prospective studies are recommended.

The robustness of the T2* segmental approach had been previously established by proper validation that documented good intra- and inter-observer, inter-study and inter-scanner reproducibility, however decreasing for higher T2* values [8, 24]. The present study showed a superior reproducibility for segmental and global T1 in the whole range of values, although T1 images showed lower contrast-to-noise-ratio.

An association between mid-septum T1 and T2* values at 1.5 T has been previously shown in relatively large cohorts of β-TM patients [14–17], and it has been postulated that T1 may provide improved myocardial tissue characterization, due to the presence of a considerable group of patients with normal T2* but low T1. However, all published studies used a value of 20 ms as T2* cutoff, established at an early stage of T2* technique development, using data from only 15 healthy subjects and an obsolete multibreathold gradient-echo technique [4]. This is a conservative cut-off that prioritizes specificity over sensitivity. In the present study, we applied a study-specific cut-off for segmental and global T2* values and the same healthy population was used to derive the lower limits of normal for native T1, highly specific to the local set-up [35].

In the present study, T2* and T1 showed a good correlation in identifying iron by a segmental approach, with 74% concordance. In 33 patients, T1 mapping could not detect iron in one or more segments, probably due to the presence of diffuse fibrosis, which is known to increase the native T1 [36]. In fact, an increased ECV, a validated surrogate marker of diffuse fibrosis [37], was detected in 16 out of the 19 discordant segments in which the ECV was available. Unlike T2*, T1 is less specific for iron, and early iron may therefore be missed in patients with diffuse fibrosis, frequent in thalassaemia patients with a history of MIO [23]. A pathologic T1 value was detected in presence of a normal T2* value for 526 segments, suggesting a higher sensitivity of T1 mapping in comparison to the T2* technique in borderline patients. In fact, in this study this discrepancy could not be explained by the use of a low-sensitive T2* threshold or by the presence of susceptibility artifacts, corrected by the use of a validated correction map [28]. Segmental variability due to susceptibility artifacts masks T2* values heterogeneity associated with inhomogeneous iron overload in patients with global T2* > 32 ms [38]. One of the most plausible explanation for the lower sensitivity of the T2* technique in the detection of mild MIO is that, due to technical constraints (maximum TE), T2* quantification loses accuracy and precision for cardiac T2* values > 20 ms [13]. Moreover, T1 and T2* seem sensitive to different form of iron. Excess iron is first stored within ferritin and, as its concentration increases further, it is gathered within hemosiderin. The T2* is predominately determined by hemosiderin [39].

The second objective of our study was to correlate global T1 measurements with demographics, CMR findings, and cardiac complications.

In healthy subjects, native T1 is influenced by gender, with women having a higher value [40]. In β-TM patients we detected comparable T1 among males and females, likely because iron masks gender differences of T1. This finding is in agreement with the T2* studies indicating that males and females are at the same risk of accumulating iron in their hearts [29, 41]. A significant inverse, although weak, association was detected between T1 and age, suggesting that MIO is an age-dependent process as previously demonstrated by the T2* technique [22].

We did not detect any correlation between native T1 and LV systolic function, volumes, or mass, probably because the majority of our patients had normal or mild abnormal values of these parameters, thanks to a good transfusional regimen. Conversely, although not frequent, LV movement abnormalities were associated with low native T1.

TM patients with replacement myocardial fibrosis had significantly lower T1. The presence of replacement myocardial fibrosis detected by the LGE technique was shown to significantly increase native T1 [42], even in non-ischemic myocardial diseases. Therefore, our finding has two important implications. Firstly, it suggests the dominance of iron over myocardial fibrosis, likely due to the small percentage of replacement fibrosis within the LV myocardium and its patchy distribution, as previously demonstrated for the T2*-replacement fibrosis relashionship [30]. Second, it suggests an association between MIO and replacement fibrosis, previously demonstrated in pediatric TM patients [43]. We introduced a threshold of 898 ms for prediction of LGE with high specificity. Conversely, the low sensitivity of our threshold (about 36%) may reflect as hepatitis C virus infection or diabetes are proved factors in the pathogenesis of replacement myocardial fibrosis in patients with β-TM [44, 45].

Finally, we here demonstrated for the first time an association between decreased native global T1 and a history of cardiac complications and we introduced a threshhold of 931 ms for the prediction of cardiac complications with satisfying sensitivity and specificity. Global myocardial T2* was similar among patients without and with cardiac complications. Likely in our population of well treated patients, generally not heavily loaded at the cardiac level, T1 emerge as a more sensitive marker of cardiac complications, stronger than global T2*. The comparison of cardiac T1 values between patients without and with active cardiac complications remained significant despite the low number of patients with active cardiac complications and despite the active complications were represented by supraventricular arrhythmias in 75% of the cases. MIO was shown to contribute less to the development of supraventricular arrhythmias than to HF [11, 12, 29].

Our findings suggest that, due to its added diagnostic value, native T1 should be included in the routine CMR assessment of β-TM patients, where available and after the definition of reference ranges for T1 values in healthy human myocardium. Such a more sensitive approach in quantifying iron could support clinicians in earlier adjustment of chelation therapy and possibly further improve prognosis in β-TM.

### Limitations

Altough the relaxation rates (R2* and R1) are proportional to the tissue iron content, we used their inverses (T2* and T1), since they are commonly adopted in the clinical practice, however an appropriate statistical approach has been used.

White blood sequences were used for T2* assessment, although black blood sequences are more accurate and robust [46].

Conversely to the T2* technique [9, 10], T1 mapping lacks appropriate calibration against histological samples.

Multicenter studies are needed to test the transferability for the T1 mapping as robust and useful complementary tool to T2* in the clinical practice and to verify the results in populations with lower prevalence of HCV infection in which a lower frequency of myocardial fibrosis may be attended.

This is a cross-sectional study and prospective studies are recommended to evaluate the predictive value of native T1 for cardiac outcomes in β-TM.

## Conclusion

Native T1 is less specific than T2* mapping for MIO detection, given that myocardial fat and diffuse fibrosis can influence T1 values. Conversely, native T1 by a segmental approach can improve the sensitivity in detecting myocardial iron loading and is associated with cardiac complications in β-TM. The combined use of both T1 and T2* mapping by a segmental approach could therefore improve the detection of mild iron loading, potentially supporting clinicians in appropriately and timely modifying chelation therapy.

## Data Availability

The datasets used and/or analysed during the current study are available from the corresponding author on reasonable request.

## Abbreviations

β-TM: Beta thalassaemia major
CI: Confidence intervals
CMR: Cardiovascular magnetic resonance
CoV: Coefficient of variation
E-MIOT: Extension-myocardial iron overload in thalassaemia
ECG: Electrocardiogram
ECV: Extracellular volume
HF: Heart failure
ICC: Intraclass correlation coefficient
LGE: Late gadolinium enhancement
LV: Left ventricle/left ventricular
MIO: Myocardial iron overload
MOLLI: Modified Look-Locker inversion recovery
OR: Odds ratio
ROC: Receiver operator curve
SD: Standard deviation
TE: Echo time
